# SLC7A11 as a Gateway of Metabolic Perturbation and Ferroptosis Vulnerability in Cancer

**DOI:** 10.3390/antiox11122444

**Published:** 2022-12-11

**Authors:** Jaewang Lee, Jong-Lyel Roh

**Affiliations:** 1 Department of Otorhinolaryngology-Head and Neck Surgery, CHA Bundang Medical Center, CHA University, Seongnam 13496, Republic of Korea; 2 Department of Biomedical Science, General Graduate School, CHA University, Seongnam 13496, Republic of Korea

**Keywords:** SLC7A11, cysteine, ferroptosis, redox, cancer

## Abstract

SLC7A11 is a cell transmembrane protein composing the light chain of system xc^−^, transporting extracellular cystine into cells for cysteine production and GSH biosynthesis. SLC7A11 is a critical gateway for redox homeostasis by maintaining the cellular levels of GSH that counter cellular oxidative stress and suppress ferroptosis. SLC7A11 is overexpressed in various human cancers and regulates tumor development, proliferation, metastasis, microenvironment, and treatment resistance. Upregulation of SLC7A11 in cancers is needed to adapt to high oxidative stress microenvironments and maintain cellular redox homeostasis. High basal ROS levels and SLC7A11 dependences in cancer cells render them vulnerable to further oxidative stress. Therefore, cyst(e)ine depletion may be an effective new strategy for cancer treatment. However, the effectiveness of the SLC7A11 inhibitors or cyst(e)inase has been established in many preclinical studies but has not reached the stage of clinical trials for cancer patients. A better understanding of cysteine and SLC7A11 functions regulating and interacting with redox-active proteins and their substrates could be a promising strategy for cancer treatment. Therefore, this review intends to understand the role of cysteine in antioxidant and redox signaling, the regulators of cysteine bioavailability in cancer, the role of SLC7A11 linking cysteine redox signaling in cancer metabolism and targeting SLC7A11 for novel cancer therapeutics.

## 1. Introduction

Glutathione is the most abundant intracellular antioxidant small molecule com-posed of three amino acids glutamate, glycine, and cysteine. Glutathione can prevent damage to vital components of cells by reactive oxygen species (ROS), such as free radicals, peroxides, lipid peroxides, and metals [1]. Glutathione exists in reduced and oxidized states. The oxidized glutathione disulfide (GSSG) is converted into two molecules of reduced glutathione (GSH) through a nicotinamide adenine dinucleotide phosphate (NADPH)-dependent reaction. In healthy cells and tissues, more than 90% of the total glutathione pool is in reduced form, and a decrease in the GSH to GSSG ratio is indicative of oxidative stress [2]. As the GSH metabolic system has been considered a potential anticancer target in many human cancers, GSH depletion can induce cancer cell death [3,4]. Over the last decade, the GSH antioxidant system has been spotlighted for its ability to reduce lipid peroxides. GSH depletion is responsible for iron-catalyzed, lipid peroxidation-dependent, non-apoptotic cell death, known as ferroptosis [5,6,7]. The induction of ferroptosis by GSH depletion has been shown to selectively kill resilient cancer cells resistant to conventional treatments in various types of human cancers [8,9].

**Figure 1:**
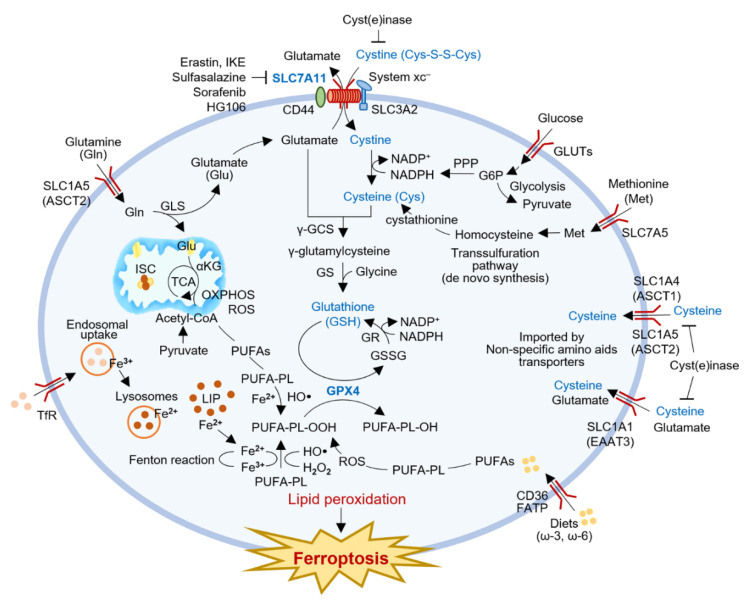
Role of SLC7A11-GSH-GPX4 axis in redox signaling and ferroptosis induction. SLC7A11 is the primary transporter for cystine import combined with glutamate export. GSH is the most abundant intracellular antioxidant composed of three amino acids; glutamate, glycine, and cysteine. Cystine uptake is essential for intracellular cysteine production and GSH biosynthesis. Cysteine may also be generated partly de novo via the transsulfuration pathway through a reduction reaction consuming NADPH or other non-specific amino acid transporters. GSH is a cofactor of GPX4, contributing to the detoxification of lipid peroxides into lipid alcohols. Therefore, GSH depletion is responsible for iron-catalyzed, lipid peroxidation-dependent, non-apoptotic cell death, known as ferroptosis. The Fenton reaction is the reaction between ferrous iron and hydrogen peroxide to form hydroxyl or peroxyl radicals that react with membrane lipids and rapidly propagate to neighboring PUFA-PL. Excessively produced lipid peroxidation disrupts the integrity of cell membranes, resulting in cell death. α-KG, α-ketoglutarate; ASCT1/2, alanine-serine cysteine transporters 1 and 2; CoA, coenzyme A; γ-GCS, γ-glutamylcysteine synthetase; GLS, glutaminase; GLUTs, glucose transporters; GPX4, glutathione peroxidase 4; GR, glutathione reductase; GS, glutathione synthetase; GSH, glutathione; GSSG, glutathione disulfide; HO·, hydroxyl radical; IKE, imidazole ketone erastin; ISC, iron-sulfur cluster; LIP, labile iron pool; NADPH, nicotinamide adenine dinucleotide phosphate; OXPHOS, oxidative phosphorylation; PPP, pentose phosphate pathway; PUFAs, polyunsaturated fatty acids; PUFA-PL, polyunsaturated fatty acid-containing phospholipid; PUFA-PL-OH, polyunsaturated fatty acid-containing phospholipid alcohol; PUFAs, polyunsaturated fatty acids; SCD, stearoyl-CoA desaturase; ROS, reactive oxygen species; SLC7A11 (xCT), solute carrier family 7 member 11; system xc^−^, cystine/glutamate exchange transporter; TCA, tricarboxylic acid cycle; Tfr, transferrin receptor.

Glutathione is produced through the two-step synthesis of a tripeptide L-glutamic acid, cysteine, and glycine (Figure 1). Cysteine required for GSH synthesis is obtained through cystine uptake through a cystine/glutamate exchange transporter, system xc^−^. Solute carrier family 7 member 11 (SLC7A11, also called xCT) is the primary transporter for cystine uptake combined with glutamate export, and plays a pivotal role in intracellular cysteine balance and GSH biosynthesis [10]. SLC7A11 inhibition blocks cysteine production and GSH biosynthesis, which can induce ferroptosis by excessive accumulation of lipid peroxidation [7]. Cysteine may be generated partly de novo via the transsulfuration pathway or other non-specific amino acid transporters [11]. Cancer cells require large amounts of cysteine and GSH to neutralize the increased intracellular ROS, and the nutrient dependency generally needs to be the increased function of SLC7A11 [12,13]. SLC7A11 overexpression is found in many human cancers and is highly sensitive to selective inhibition of SLC7A11 [14]. The distinct anticancer effect of SLC7A11–GSH axis blocking has been established in various human cancers [15]. A better understanding of cysteine and SLC7A11 functions regulating and interacting with redox-active proteins and their substrates could be a promising strategy for cancer treatment. Therefore, this review intends to understand the role of cysteine in antioxidant and redox signaling, the regulators of cysteine bioavailability in cancer, the role of SLC7A11 linking cysteine redox signaling in cancer metabolism and targeting SLC7A11 for novel cancer therapeutics.

## 2. Role of Cysteine in Antioxidant and Redox Signaling

Cysteine (2-amino-3-mercaptopropionic acid) is a naturally occurring sulfur-containing semi-essential or conditionally essential amino acid with the formula HOOC−CH(−NH_2_)−CH_2_−SH. Cysteine was named after cystine, derived from the Greek word kustis, meaning bladder because cystine was first isolated from kidney stones. Cysteine is the only one of the 20 standard amino acids to contain a thiol group (-SH). The thiol group undergoes a reversible redox reaction when cysteines are oxidized to form cystine through sulfur bonds between the two cysteines. Conversely, the reduction of cystine forms two cysteines. The sulfur bond of cystine is vital in the determination of many protein structures and aids in the catalysis of enzymes [16]. The thiol group of cysteine is also nucleophilic and can therefore be involved in nucleophilic addition and substitution reactions [17]. The cysteine residues of proteins are close to neutral, but the thiol groups become more active when ionized in cells. Cysteine is a major source of sulfur in human metabolism by creating a sulfur bond with other thiol groups when oxidized in cells [18]. Cysteine has antioxidant properties because of its ability to participate in redox molecular switching [19]. Cysteine is indispensable for the biosynthesis of essential metabolites involved in various biological processes, e.g., iron-sulfur clusters and coenzyme A (CoA).

Cysteine is an essential precursor to producing antioxidant GSH in the human body and other tissues. The effect of GSH oral administration is minimal, and most GSH is produced in cells [20]. As a major antioxidant tripeptide composed of glycine, glutamic acid, and cysteine, glycine and glutamic acid are readily available in the diet, but the uptake rate of cysteine is a limiting factor for intracellular GSH synthesis. The thiol group of cysteine acts as a proton donor, which contributes to the biological activity of GSH [17]. Cysteine is potentially toxic and unstable in a highly oxidizing extracellular environment [21]. Thus, cysteine is absorbed in the form of cystine because it is more stable in the digestive tract and plasma. Cystine safely travels through the gastrointestinal tract and plasma and is broken down into two cysteines as it enters cells via the cystine/glutamate antiporter system xc^−^ [21]. Cystine imported in cells is converted into cysteine in the cytosol through a reduction reaction consuming NADPH generated by the hexose monophosphate shunt, an alternative pathway of glucose metabolism [22]. Intracellular cysteine can also be synthesized de novo, from methionine and serine via the transsulfuration pathway [11]. Cysteine is a precursor or cofactor of other antioxidant biomolecules, such as taurine, hydrogen sulfide, and aconitic acid [23].

Cysteine participates in the biosynthesis of GSH through two steps and is a rate-limiting precursor of GSH synthesis. Cysteine and glutamate synthesize γ-glutamylcysteine (GGC) by glutamate cysteine ligase (GCL), and glycine is added to its C-terminus by glutathione synthetase (GS) to form GSH. GSH is a cofactor of glutathione peroxidase 4 (GPX4), contributing to the detoxification of lipid peroxides into lipid alcohols [24]. Oxidized GSSG is reduced to GSH by consuming H^+^ from NADPH by glutathione reductase (GR), enabling GSH recycling. In general, the GSH-to-GSSG ratio determines the redox state of a cell [25]. The GSH/GSSG ratio is higher in a more reduced environment where cell proliferation is active, and the ratio is lower in a more oxidized environment associated with differentiation. The GSH/GSSG ratio also differs among intracellular organelles; higher in a reducing environment in the cytoplasm and lower in an oxidizing environment in the endoplasmic reticulum. GSH is a pivotal regulator of ferroptosis, a newly discovered form of iron-dependent regulated cell death induced by lipid peroxidation. GSH is a cofactor of GPX4 that inhibits lipid peroxidation and limits ferroptosis [5,6,7]. Therefore, a deficiency of intracellular cysteine and GSH can promote lipid peroxidation and induce ferroptosis.

## 3. Regulators of Cysteine Bioavailability in Cancer Cells

The bioavailability of cysteine in cancer cells can affect the fitness of cellular metabolism and the development of treatment resistance. Cellular cysteine can be mainly acquired by cystine uptake from extracellular sources. Cysteine can also be produced by extracellular GSH catabolism, protein catabolism, and de novo synthesis from methionine via the transsulfuration pathway. However, their supply is insufficient to meet the high demand for antioxidant defense in cancer cells and is more likely transient in cancer cells that encounter intermittent cyst(e)ine deficiency [11]. Therefore, most cancer cells depend on the supply of cysteine from the extracellular environment via nutrient transporters. The majority of cellular cysteine is produced by dietary intake of cystine, the oxidized form of cysteine. The oxidative environment of plasma promotes cystine formation by dimerizing cysteine and allows uptake into cells from the surrounding milieu. Intracellular cystine uptake is achieved through the system xc^−^, which imports extracellular cystine while exporting intracellular glutamate at a ratio of 1:1 [17]. The system xc^−^ consists of two subunits: heavy chain (SLC3A2; also called CD98 or 4F2hc) and light chain (SLC7A11) solute carrier family [26]. SLC7A11 comprises 12 highly hydrophobic channel transmembrane proteins with both N- and C-termini located in the cytoplasm. SLC3A2, a single transmembrane protein, is a chaperone that helps the stability and appropriate membrane location of the SLC7A11 protein.

The mRNA expression of SLC7A11 differs in each tissue of the human body: it is highest in the central nervous system (CNS) and some cells of the immune system, such as antigen-presenting cells (APCs) and myeloid-derived suppressor cells (MDSCs), and is relatively low in the kidney, heart, and liver [27]. Notably, SLC7A11 overexpression is observed in various human cancers, including lymphoma, leukemia, squamous cell carcinoma, breast cancer, glioblastoma, and pancreatic ductal adenocarcinoma (PDAC) [15]. In the CNS, extracellular glutamate moves into cells to form synaptic vesicles, and an imbalance in cellular glutamate homeostasis can cause psychosis, neurodegenerative diseases, and brain cancers [28]. In addition, as cancers grow, it is essential to maintain cellular GSH levels by reducing cysteine to maintain the balance of redox systems in cancer cells. Cysteine is necessary for cancer cell survival as it maintains the levels of GSH required for cell growth and proliferation, redox cycling, antioxidant defense, detoxification, and immune responses. Therefore, overexpression of SLC7A11 expression for activation of cystine uptake is observed in various types of human cancers [14]. Cysteine can also be imported from the extracellular milieu into cells by non-specific transporters, such as excitatory amino acid transporter 3 (EAAT3) and the alanine-serine cysteine transporters 1 and 2 (ASCT1/2). These transporters are also associated with transporting other amino acids, e.g., glutamine and glutamate. A limited number of studies have examined these transporters in cancer: their overexpression has been observed in different cancer cells and associated with increased chemoresistance in colorectal and prostate cancers [29].

**Figure 2:**
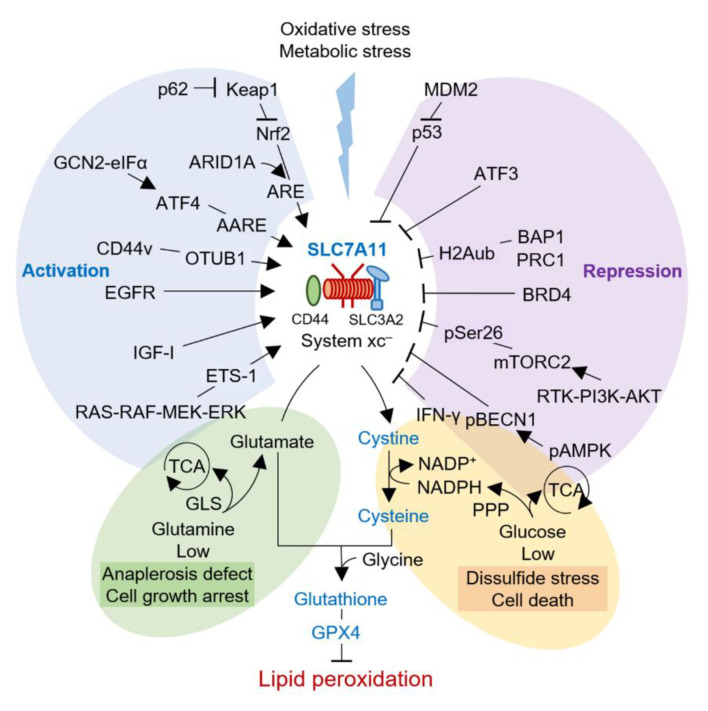
Regulators of the SLC7A11–cysteine–GSH axis in cancer cells. Cellular cysteine is mainly acquired by cystine uptake from extracellular sources via SLC7A11. SLC7A11 expression can be activated or repressed via various regulatory mechanisms at the transcription, translation, and posttranslational levels. Cancer cells require large amounts of cysteine and GSH to neutralize the increased intracellular ROS, and the nutrient dependency generally needs the increased function of SLC7A11. These cancer cells may be rendered more susceptible to limiting glucose or glutamine supply. SLC7A11 overexpression is found in many human cancers and is highly sensitive to selective inhibition of SLC7A11 or cyst(e)inase. AARE, amino acid response elements; AMPK, AMP-activated protein kinase; ARE, antioxidant response element; ATF4, activating transcription factor 4; BAP1, BRCA1-associated protein-1; BRD4, bromodomain-containing protein 4; CD44v, CD44 variant; EAAT3, excitatory amino acid transporter 3; EGFR, epidermal growth factor receptor; GCN2-eIF2α, general control non-derepressible-2-eukaryotic initiation factor 2α; IFN-γ, interferon-gamma; IGF-I, insulin-like growth factor-I; Keap1, Kelch-like ECH-associated protein 1; MDM2, murine double minute 2; mTORC2, mammalian target of rapamycin complex 2; Nrf2, nuclear erythroid 2-related factor; OTUB1, ubiquitin hydrolase otubain-1; pBECN1, phospho-beclin-1.

The regulatory mechanism of SLC7A11 is mapped out at the DNA, transcription, translation, and posttranslational levels. Activating transcription factor 4 (ATF4) and nuclear erythroid 2-related factor 2 (Nrf2) are two major players in stress-induced SLC7A11 transcription (Figure 2).

ATF4 is involved in redox homeostasis, amino acid metabolism, and endoplasmic reticulum stress to promote the transcription of SLC7A11. Amino acid starvation, endoplasmic reticulum stress, and hypoxia may increase ATF4 mRNA translation through the general control non-derepressible-2 (GCN2)-eukaryotic initiation factor 2α (eIF2α) signaling axis [30]. Consequently, ATF4 binds to the amino acid response elements (AARE) and promotes the transcription of genes involved in stress response, including SLC7A11, thereby enabling cells to cope with amino acid deficiency [30]. ATF4 promotes ferroptosis resistance in cancer cells by upregulating SLC7A11 as an adaptive response to cystine deficiency [31]. The Kelch-like ECH-associated protein 1 (Keap1)-Nrf2-activator protein-1 (AP-1)/antioxidant response element (ARE) signaling pathway increases the transcription of genes responsible for resistance to oxidative stress, including SLC7A11 [32]. Nrf2 is unstable under basal conditions and is ubiquitinated by an E3 ubiquitin ligase Keap1. Oxidative stress impairs Nrf2 degradation by Keap1 and allows Nrf2 to bind to ARE, which is involved in antioxidant defense and redox maintenance. In cancer cells, Keap1 inactivation promotes ferroptosis resistance following SLC7A11/cysteine/GSH axis activation by stabilizing Nrf2 and Nrf2 target genes [33]. Nrf2 may also mediate ferroptosis resistance by regulating genes associated with GSH biosynthesis, iron metabolism, and antioxidant responses [34]. SLC7A11 expression can also be repressed by transcription factors that play a role in tumor suppression. p53 directly represses the transcription of SLC7A11 and inhibits ferroptotic cell death by various ferroptosis inducers [35]. ATF3, a common stress sensor, binds to the SLC7A11 promoter under basal conditions and represses its expression independent of p53. Erastin treatment or cystine deficiency induces SLC7A11 expression, but upregulating ATF3 suppresses SLC7A11 expression, depletes intracellular GSH, and promotes ferroptosis in cancer cells [36].

Transcription of SLC7A11 can also be regulated by epigenetic modifications on DNA and/or DNA-associated histones, such as acetylation, methylation, ubiquitination, and phosphorylation [37]. BRCA1-associated protein-1 (BAP1) is a nuclear protein that removes histone H2A monoubiquitylation (H2Aub) at the 119 position of lysine and inhibits transcription of SLC7A11 [38]. Deubiquitinating H2Aub on the SLC7A11 gene promoter represses its expression, inhibiting cystine uptake and GSH synthesis and promoting ferroptosis in cancer cells. Methylation of histone H3 (H3K9me3 and H3K27me3) also contributes to the transcriptional repression of SLC7A11. Bromodomain-containing protein 4 (BRD4) recognizes acetylated histones and recruits transcription factors, which can inhibit the transcription of SLC7A11 [39]. A recent study revealed that ARID1A, which encodes a component of the switch/sucrose non-fermentable (SWI/SNF) chromatin-remodeling complex, could promote Nrf2-mediated transcriptional activation of SLC7A11 [40]. SWI/SNF deficiency inhibits SLC7A11 transcription, impairs cystine uptake and GSH biosynthesis, and promotes lipid peroxidation-induced ferroptosis in cancer cells. In addition, the adhesion molecule CD44 variant (CD44v) forms a complex that binds to SLC7A11, maintaining the stability of SLC7A11 [41]. CD44 expression enhances the stability of SLC7A11 and suppresses ferroptosis by promoting the direct interaction between the ubiquitin hydrolase otubain-1 (OTUB1) and SLC7A11. CD44v depletion partially abrogates this interaction, induces SLC7A11 inactivation, and promotes ferroptosis in cancer cells. Mammalian target of rapamycin complex 2 (mTORC2) also inhibits the activity of SLC7A11 by directly phosphorylating SLC7A11 at serine 26 through the AKT signaling pathway [42]. A key autophagy regulator, Beclin-1 (BECN1), represses system xc^−^ activity through direct binding to SLC7A11 and thereby involves lipid peroxidation and ferroptosis induction [43]. The BECN1-induced ferroptosis requires AMP-activated protein kinase (AMPK)-mediated phosphorylation of BECN1 at Ser90/93/96 when cancer cells are exposed to system xc^−^ inhibitors, e.g., erastin, sulfasalazine, and sorafenib. Inhibition of mTOR promotes glutamate secretion, cystine uptake, and GSH biosynthesis, enabling cancer cells to adapt to rapidly changing environments. The epidermal growth factor receptor (EGFR) may also interact with SLC7A11 and maintain its proper localization on the plasma membrane [44]. EGFR-expressing glioma cells exhibit increased glutamate export, cystine uptake, and GSH biosynthesis, while targeted inhibition of SLC7A11 suppresses the antioxidant capacity, growth, and invasion of EGFR-overexpressing cancer cells. Insulin-like growth factor-I (IGF-I) regulates cystine uptake and redox status in ER+ breast cancer cells by activating SLC7A11 expression [45]. In summary, these emerging studies have shown that diverse posttranslational mechanisms of SLC7A11 can modulate protein stability, localization, and transporter activity.

## 4. Role of SLC7A11 Linking Cysteine Redox Signaling to Cancer Metabolism

Cysteine is mainly produced by cystine uptake into cells through the SLC7A11 subunit of system xc^−^. Cancer cells critically depend on the intracellular uptake of amino acids from their microenvironments, and extracellular cystine uptake is required for cancer growth and progression [46]. Cancer cells are hallmarked by resistance from cell death, most notably apoptosis [47]. The characteristics make precancerous cells or cancer cells exposed to metabolic stress or nutritional deficiencies resistant to apoptosis or other types of cell death. Recent studies have unraveled that ferroptosis, similar to apoptosis, is actively involved in the mechanisms of inhibiting tumorigenesis in changing microenvironments [12]. SLC7A11 is involved in antioxidant defense and cellular redox homeostasis through cysteine and GSH production and has emerged as a central hub linking its ferroptosis suppression to tumor initiation and progression.

### 4.1. SLC7A11 Promotes Tumorigenesis via Inhibiting Ferroptosis

Cellular redox balance plays a critical role in cellular transformation and tumorigenesis through redox homeostasis between mutagenic ROS production and tight control by antioxidant programs responsive to cellular stressors [48,49]. Enhanced intracellular GSH biosynthesis by SLC7A11 overexpression is essential for oncogenic RAS transformation by protecting cells from oxidative stress and cell death [50]. Transcriptional upregulation of SLC7A11 results from the ETS-1 transcription factor downstream of the RAS-RAF-MEK-ERK signaling cascade, directly transactivating the SLC7A11 promoter in synergy with ATF4. Notably, genetic depletion or pharmacological inhibition of SLC7A11 induces synthetic lethality in KRAS-mutant lung adenocarcinoma, highlighting SLC7A11 as a potential therapeutic target for RAS-driven tumors [51]. Interestingly, sulfasalazine and HG106 induce the selective inhibition of SLC7A11, but both drugs exhibit different types of cell death by increasing cellular oxidative stress, namely ferroptosis and apoptosis, respectively. This suggests that SLC7A11 may have different functions independent of ferroptosis in promoting tumor development, such as apoptosis and other non-ferroptotic cell death. OTUB1 deubiquitinates and stabilizes the SLC7A11 protein by direct interaction [41]. OTUB1 overexpression is frequently found in various human cancers, which maintains high expression of SLC7A11 in cancer cells by posttranslational regulation of OTUB1.

De-repression of SLC7A11 also promotes tumor development partly via inhibiting ferroptosis, e.g., genetic mutations or deletions of tumor suppressor p53 or BAP1. p53 is the most frequently mutated tumor suppressor in human cancers, suggesting that the p53-induced transcriptional repression of SLC7A11 plays an important role in p53-mediated tumor suppression [52]. The p53 mutation at three acetylation sites (K117R+K161R+K162R, 3KR mutant) loses its ability to induce cell cycle arrest, senescence, and apoptosis, yet still is capable of regulating ROS production and suppressing tumor formation [53]. The preserved function of tumor suppression in the p53 3KR mutant has been later unraveled, partly by repressing SLC7A11 expression [35]. However, the additional mutation (K98R) in the p53 3KR mutant markedly abolishes the ability of p53 to suppress tumor formation by repressing SLC7A11 expression and inducing ferroptosis in cancer cells [54]. Arachidonate 12-lipoxygenase (ALOX12) also plays a critical role in p53-mediated ferroptosis [55]. ALOX12 mediates polyunsaturated fatty acid (PUFA) peroxidation and ferroptosis independently of the canonical ferroptosis pathway through the GPX4 and acyl-CoA synthetase long-chain family member 4 (ACSL4) axis. Mechanistically, SLC7A11 interacts with ALOX12, which suppresses PUFA peroxidation and ferroptosis. ALOX12 mutations in human cancers promote tumorigenesis by abrogating its ability to oxygenate PUFAs and induce ferroptosis. BAP1 is another tumor suppressor repressing SLC7A11 transcription through H2A histone ubiquitination, which inhibits cystine uptake and GSH biosynthesis, and promotes ferroptosis [38]. As BAP1 is frequently mutated in human cancers, BAP1 mutation contributes to tumor development by abrogating its ability to suppress the SLC7A11 expression and induce ferroptosis [56].

### 4.2. SLC7A11 Promotes Immune Evasion, Invasion, and Metastasis in Human Cancers

In the tumor microenvironment, SLC7A11 is involved in tumor survival and proliferation through the interaction between immune cells and tumor cells. Interferon gamma (IFN-γ) secreted by CD8^+^ cytotoxic T cells promotes lipid peroxidation and ferroptosis by inhibiting the expression of SLC3A2 and SLC7A11, two subunits of system xc^−^ in tumor cells [57]. Cysteine is an essential amino acid for T-cell activation. T-cells lacking SLC7A11 or cystathionases rely on neutral amino acid transporters to release cysteine from APCs [58]. Cysteine export by APCs is reduced by the presence of MDSCs, limiting antitumor immunity by T-cell activation [59]. In glioma cells, an increase in extracellular glutamate caused by overexpression of SLC7A11 impairs cytotoxic T-cell activation and promotes regulatory T (Treg)-cell proliferation, leading to intratumoral immunosuppression [60,61]. The altered cancer metabolism through overexpression of SLC7A11 promotes immune evasion of glioblastoma multiforme (GBM) through dysfunction of T cell activation. Antitumor immunity caused by T cell activation is also diminished by CD36-mediated uptake of fatty acids in tumor-infiltrating CD8^+^ T cells that induces lipid peroxidation and ferroptosis of the cells [62]. Additionally, SLC7A11 has a potential role in cancer-associated fibroblasts (CAFs) or vascular remodeling. SLC7A11 is highly expressed in CAFs, enabling tumor cells to protect against exogenous oxidative stress [63]. In human cancer, ATF4 promotes the transcription of genes involved in stress response, including SLC7A11, to increase tumor angiogenesis and shape blood vessel architecture [31].

Increased expression of SLC7A11 and/or CD44 is found in various human cancers and is closely associated with tumor invasion, lymph node metastasis, recurrence, and poor prognosis [64,65]. SLC7A11-mediated glutamate release promotes glioma cell infiltration and could be blocked by xCT inhibitors such as sulfasalazine and (S)-4-carboxyphenylglycine [66]. SLC7A11 expression is also involved in the invasion and metastasis of melanoma, and loss of SLC7A11 can inhibit melanoma metastasis in vivo [67]. PDAC has a highly metastatic potential with few effective therapeutic options. Mitochondrial calcium uniporter (MCU) can promote tumor metastasis by activating the Keap1–Nrf2–SLC7A11 axis [68]. SLC7A11 inhibition in MCU-high PDAC effectively induces tumor regression and abolishes MCU-driven metastasis. In addition, CAF highly depends on cystine uptake and GSH synthesis via SLC7A11 expression in PDAC. Therefore, targeting SLC7A11 in both compartments of PDAC stromal and tumor cells could be a more effective treatment approach [63].

SLC7A11-mediated extracellular glutamate secretion can also promote the intrinsic invasiveness of cancer cells. Glutamate release by SLC7A11 promotes tumor invasion through the upregulation of membrane type 1 metalloprotease and basement membrane disruption in breast cancer cells [69]. Glutamate excretion by IL-1β-induced SLC7A11 overexpression can also promote hepatoma metastasis through the upregulation of programmed death ligand 1 (PD-L1) and colony-stimulating factor 1 (CSF1) [70]. Pharmacological interference of glutamate release from tumor cells can limit host bone response and impairs bone metastasis of cancer cells [71].

### 4.3. SLC7A11 Induces Nutrient Dependency and Metabolic Vulnerability in Cancer

Altered energy metabolism is a hallmark of cancer that can be an effective treatment target [72]. Tumors are metabolically diverse by reprogramming pathways for nutrient acquisition. A better understanding and detection of tumor metabolic reprogramming has been increasingly supported as a new strategy to treat human cancer. Cancer cells promote tumor growth and proliferation through amino acid metabolism reprogramming. Tumor cells maintain the redox balance and cell survival by developing antioxidant systems to control the increased cellular levels of ROS along with their proliferation [48,49].

As a major antioxidant, GSH biosynthesis requires cysteine. Cancer cells import a large amount of cystine into the cell through high levels of SLC7A11 expression (SLC7A11^high^) and quickly reduce highly insoluble cystine to more soluble cysteine. This reaction requires a cellular NADPH pool mainly drained from the glycolysis–pentose phosphate pathway [22]. Therefore, cancer cells with SLC7A11^high^ are highly dependent on this pathway and render such cells susceptible to limiting glucose supply [13,73]. Co-targeting glucose transporter type 1 (GLUT1) and GSH biosynthesis can induce NADPH depletion, marked accumulation of cystine and other disulfide molecules, and ROS accumulation, leading to the synthetic lethality of SLC7A11^high^ tumor cells [74,75]. However, SLC7A11 knockdown or pharmacological inhibition by sulfasalazine in SLC7A11^high^ cancer cells reduces cellular ROS levels and cell death induced by glucose deprivation [76]. This suggests that cellular ROS following glucose deprivation plays a critical role in SLC7A11-dependent cancer cell death. Additionally, high cell density in glioma cells promotes lysosomal degradation of SLC7A11, which may enable metabolic adaptation and cell survival [77].

SLC7A11 simultaneously imports cystine and exports glutamate at a 1:1 ratio. SLC7A11-mediated glutamate transport results in a deficiency of the intracellular glutamate-α-KG pool, requiring more glutamine uptake. This affects the nutritional dependence of cancer cells through glutamine anaplerosis and glutaminase (GLS) [78]. Cancer cells in SLC7A11^high^ or cystine-rich conditions respond sensitively to glutamine analogs or glutaminolysis inhibitors that inhibit glutamine anaplerosis to the TCA cycle [79]. However, the upregulation of SLC7A11 antagonizes glutamine metabolism and restricts nutrient flexibility despite the cellular need for antioxidant defense [80]. Therefore, cancer cells reprogram their amino acid metabolism for adaptation to the changing microenvironment of nutrition. mTORC2 is a critical regulator of amino acid metabolism in cancer and can inhibit the activity of SLC7A11 by direct phosphorylation at serine 26 [42]. In an environment lacking micronutrient levels, cancer cells can regulate the function of SLC7A11 by mTORC2-mediated phosphorylation to protect themselves from cellular stress that facilitates increasing glutamate efflux and cystine uptake [42].

Cancer cells with SLC7A11^high^ highly depend on specific amino acids, such as glucose and glutamine, which may force the establishment of a novel therapeutic strategy to target cancer-specific metabolic vulnerabilities. In SLC7A11^high^ GBM cells, glucose restriction decreases mismatch repair genes and increases double-strand breaks, making cancer cells more susceptible to radiation therapy [81]. CD44v-expressing stem-like head and neck squamous cell carcinoma (HNSCC) cells retain metabolic reprogramming toward increased glutaminolysis, which renders the cells more sensitive to xCT inhibitors with the combination of glutamate dehydrogenase (GDH) inhibition [82]. However, cystine starvation could rescue glucose starvation-induced cell death in SLC7A11^high^ cancer cells and render such cells less susceptible to ferroptosis induced by SLC7A11 inhibition [73]. In SLC7A11^high^ cancer cells, the additional supply of cysteine, such as N-acetyl cysteine (NAC), could rescue the cells from glucose starvation but not from glutamine deprivation [73,83]. Therefore, it is necessary to further underpin the mechanistic understanding of nutrient dependence in cancer cells with the SLC7A11^high^ cellular phenotype.

### 4.4. SLC7A11 Has a Role in Cancer Therapeutic Resistance

SLC7A11 expression is closely related to treatment resistance through multiple pathways such as the antioxidant system, nutritional limitation, autophagy, and multidrug resistance in cancer cells. A previous study screened the potency of 1400 candidates, including amino acid analogs, L-alanosine, and geldanamycin, with anticancer effects in 60 human cancer cell lines [84]. SLC7A11 mediated cellular uptake of L-alanosine in cancer cells and conferred chemoresistance to geldanamycin by supplying cystine for GSH biosynthesis. Therefore, the SLC7A11 expression of cancer cells can be an important target for predicting resistance to anticancer drugs and overcoming treatment resistance.

The cell adhesion molecule CD44v interacts with SLC7A11 and stabilizes the protein in the plasma membrane, thus facilitating cystine uptake into cells [41]. CD44v-mediated upregulation of SLC7A11 promotes cystine supply and GSH synthesis, thereby inducing anticancer drug resistance in cancer cells [15]. CD44v expression is associated with 5-fluorouracil resistance in gastric cancer cells and may be abolished by SLC7A11 inhibition [85]. In addition, SLC7A11 inhibition induces selective cell death in CD44v-expressing HNSCC that are intrinsically resistant to EGFR-targeted therapy [86]. High CD44v and SLC7A11 expression are closely associated with the resistance to cisplatin in liver and bladder cancers, and sulfasalazine can eradicate the chemoresistant cancer cells [87,88].

Even after chemotherapy or radiotherapy, some cancer cells upregulate the expression of SLC7A11 to resist oxidative stress, inhibit cell death, and develop treatment resistance. Nrf2 and SLC7A11 are overexpressed in esophageal cancers, contributing to resistance to radiation and ferroptosis [89]. Enhanced expression of SLC7A11 is also found in GBM cells, partly due to the activation of Nrf2 [90]. An increase in cellular ROS by gene knockdown or pharmacological inhibitors of SLC7A11 leads to a synergistic effect in antitumor therapies. In CD133-positive hepatocellular carcinoma cells, the antioxidant defense systems against ROS are enhanced and play a central role in treatment resistance [91]. Sulfasalazine may improve the effectiveness of anticancer therapies by impairing the ROS defense system.

Conversely, a recent study showed that low expression of SLC7A11 was associated with resistance to paclitaxel and a low survival rate in ovarian cancer patients. Low expression of SLC7A11 was found in 90 drug-resistant ovarian cancer cell tissues, resulting from that, SLC7A11 strongly regulated cell autophagy as a competing endogenous RNA [92]. The multidrug-resistant protein P-glycoprotein (P-gp) is one of the most important defense mechanisms for cancer cell survival against anticancer drugs. Low regulation of SLC7A11 or cystine deprivation induces ROS-induced overexpression of P-gp in breast cancer cells and drug resistance [93]. SLC7A11 overexpression or cystine supplementation strongly reduces the expression and activity of P-gp. Cystine supply or NAC treatment renders drug-resistant lung cancer cells more sensitive to anticancer drugs [94]. This suggests that ROS and SLC7A11 are major factors affecting P-gp expression and function, and SLC7A11 is a potential target for regulating P-gp-related drug resistance.

## 5. Targeting SLC7A11 for Novel Cancer Therapeutics

Ferroptosis is a recent advance in oxidative-regulated cell death induced by the accumulation of iron-mediated lipid peroxidation [6]. Iron-loaded ROS production promotes PUFA peroxidation in ferroptosis (Figure 1). The Fenton reaction is the reaction between ferrous iron and hydrogen peroxide to form hydroxyl or peroxyl radicals that react with membrane lipids and rapidly propagate to neighboring PUFA-phospholipids [95]. Excessive lipid peroxidation disrupts the integrity of cell membranes, resulting in cell death [6]. Lipid peroxidation is driven by multiple iron-containing enzymes such as arachidonate lipoxygenases, e.g., 12/15-lipoxygenase, P450 oxidoreductase, and prostaglandin-endoperoxide synthase 2 [96]. The radical-trapping antioxidant systems protect cells from the excessive accumulation of cellular ROS by reducing ROS to H_2_O. GPX4 and SLC7A11 are the essential modulators of ferroptosis [5]. GPX4 is a major cellular antioxidant that reduces lipid hydroperoxides to lipid alcohols, resulting from the oxidation of GSH. SLC7A11 is a membrane protein that contributes to detoxifying lipid peroxidation by participating in the intracellular uptake of cystine for GSH production. GPX4 requires GSH as a cofactor that inhibits lipid peroxidation, and thereby the depletion of cysteine and GSH could inactivate the protective effect of GPX4 [45].

Ferroptosis was first coined by professor Stockwell and colleagues and is attracting attention as a novel treatment method for various human diseases [5,7]. In 2012, Dixon et al., screened lethal compounds triggering specific elimination of RAS-mutated cancer cells, which led to finding a novel form of non-apoptotic cell death, ferroptosis, that was morphologically, biochemically, and genetically distinct from other types of regulatory cell death [7]. Since then, the molecular regulation of ferroptosis has been elucidated through various model studies, and the biochemical characteristics of ferroptosis could be inhibited by iron chelators or lipophilic antioxidants [5,7]. The constitutive activity of SLC7A11 inhibits ferroptosis in various cells, while gene knockdown or pharmacological inhibition of SLC7A11 could induce ferroptosis. Notably, ferroptosis by SLC7A11 inhibition can be suppressed by β-mercaptoethanol, which reduces extracellular cystine to cysteine and promotes bypass of the system xc^−^ [5]. Although SLC7A11 is overexpressed in various cancers, cancer cells maintain redox homeostasis by developing different antioxidant defenses to survive high levels of oxidative stress. Normal cells can replace SLC7A11 function by cystine uptake via additional transporters other than SLC7A11, or obtaining intracellular cysteine through de novo cysteine synthesis [97]. Cancer cells further develop the antioxidant systems necessary for oncogene adaption to induce overexpression of SLC7A11, which selectively targets cancer cells while minimizing adverse effects on normal cells [98]. SLC7A11 knockout, unlike GPX4 knockout, does not result in embryonic lethality and does not affect the development or phenotypes of the pancreas and other major organs [99,100]. Therefore, targeting SLC7A11 may be a promising therapeutic strategy to selectively treat cancer with minimal effects on normal tissues.

Several compounds have been identified as SLC7A11 inhibitors, including erastin, imidazole ketone erastin (IKE), sulfasalazine, and sorafenib [6,51]. These agents were characterized as class 1 ferroptosis inducers (FINs) capable of inducing ferroptosis by blocking cystine uptake of SLC7A11. Erastin is the most widely used class 1 FIN, which has been discovered to selectively eliminate cancer cells harboring the oncogenic mutant RAS [101]. However, erastin cannot be used in animal experiments or humans due to poor metabolic stability and low solubility in vivo [6]. IKE, an erastin analog with high metabolic stability and solubility, has nanomolar potency and suitability for testing ferroptosis in preclinical studies [102]. IKE treatment mimics the effects of cystine depletion, such as cystine starvation or system xc^−^ inhibition, which is reversed by co-treatment with iron chelators, ferrostatin-1, or NAC in cancer cells. IKE could effectively suppress the growth of pancreatic cancers vulnerable to the cystine-deprived, hypoxic microenvironment in a genetically engineered mouse model of PDAC [98]. However, IKE was developed relatively recently and has not yet moved to the clinical trial stage in cancer patients. Sulfasalazine and sorafenib are currently being actively used in clinical patients for the treatment of arthritis and human cancers, respectively, under the approval of the U.S. Food and Drug Administration. Both drugs can suppress tumor growth by inhibiting the SLC7A11 transporter activity of SLC7A11 and ferroptosis in vivo [6,103,104]. HG106, recently known as a potent SLC7A11 inhibitor, also showed marked tumor suppression and prolonged survival in the preclinical mouse models of KRAS-mutated lung adenocarcinoma [51]. Recently, an engineered and pharmacologically optimized human cyst(e)inase enzyme could suppress tumor growth in PDAC, prostate, and breast cancer xenografts [98,105]. Systemic administration of cyst(e)inase depleted serum L-cysteine and L-cystine pools and doubled the median survival time of TCL1-Tg:p53^-/-^ mice resembling chronic lymphocytic leukemia [105]. In summary, although the class 1 FIN agents have proven their effectiveness in numerous preclinical studies, the proof of concept has rarely been established in clinical trials in cancer patients. Therefore, it is urgent to develop more therapeutically effective and minimal side-effect SLC7A11 inhibitors and test them in rigorous preclinical models and clinical trials.

## 6. Conclusions and Perspectives

Cysteine is an amino acid that plays versatile roles in protein synthesis, posttranslational modification, and cystine import. Moreover, cysteine is a redox-active amino acid retaining critical antioxidant capacity by participating in redox homeostasis through GSH biosynthesis and acting as a proton donor for the biological activity of GSH. Cysteine is an essential precursor for the production of antioxidant GSH, of which the majority is synthesized in cells and is critically dependent on the uptake rate of cystine. Cysteine is mainly produced through cystine import through SLC7A11, constituting system xc– and contributes to GSH biosynthesis. GSH acts as a cofactor of GPX4 that contributes to the detoxification of lipid peroxides into lipid alcohols, and the SLC7A11–GSH–GPX4 axis is known as the canonical pathway of ferroptosis. Therefore, the cysteine–GSH axis is a pivotal regulator of ferroptosis, a new form of iron-catalyzed regulatory cell death by excessively accumulating lipid peroxidation.

SLC7A11 plays a vital role in regulating cellular redox status by countering cellular oxidative stress and suppressing ferroptosis through cystine import and GSH synthesis. SLC7A11 is overexpressed in various types of human cancers and is deeply involved in regulating tumor development, proliferation, metastasis, microenvironment, and treatment resistance. Many cancer cells depend more on the GSH antioxidant system to maintain cellular redox balance from high intrinsic oxidative stress. SLC7A11 is a critical gateway for cellular redox homeostasis through its regulation of the pathway leading to cystine import, cysteine production, and GSH biosynthesis, SLC7A11 overexpression in many cancer cells renders them susceptible to abrogation of SLC7A11, and thereby cyst(e)ine depletion may be an effective new strategy for cancer treatment. Some cancer cells, such as lymphocytic leukemia cells, cannot synthesize cysteine by other pathways and are highly dependent on the uptake of extracellular cystine to maintain intracellular GSH levels in large amounts at millimolar concentrations. High basal ROS levels and SLC7A11 dependence in cancer cells render them vulnerable to further oxidative stress, thereby making them highly sensitive to SLC7A11 inhibition. SLC7A11 inhibitors or cyst(e)inase that deplete extracellular cyst(e)ine could be a promising strategy to overcome cancer treatment resistance. However, although the effectiveness of the SLC7A11 inhibitors or cyst(e)inase has been established in many preclinical studies, these have not reached the stage of clinical trials for cancer patients. Therefore, it is highly anticipated that more effective new SLC7A11 inhibitors and targeting methods will be developed and confirmed not only in preclinical models of various cancers but also in the clinical trials of cancer patients shortly.

## Data Availability

The data are contained within the article.

## Abbreviations

AARE, amino acid response elements; ACSL4, acyl-CoA synthetase long-chain family member 4; ALOX12, arachidonate 12-lipoxygenase; AMPK, AMP-activated protein kinase; AP-1, activator protein-1; APCs, antigen-expressing cells; ARE, antioxidant response element; ASCT1/2, alanine-serine cysteine transporters 1 and 2; ATF4, activating transcription factor 4; BAP1, BRCA1-associated protein-1; BRD4, bromodomain-containing protein 4; CAFs, cancer-associated fibroblasts; CD44v, CD44 variant; CoA, coenzyme A; EAAT3, excitatory amino acid transporter 3; EGFR, epidermal growth factor receptor; FINs, ferroptosis inducers; GBM, glioblastoma multiforme; GCL, glutamate cysteine ligase; γ-GCS, γ-glutamylcysteine synthetase; GDH, glutamate dehydrogenase; GGC, γ-glutamylcysteine; GLS, glutaminase; GLUT1, glucose transporter type 1; GPX4, glutathione peroxidase 4; GR, glutathione reductase; GS, glutathione synthetase; GSH, glutathione; GSSG, glutathione disulfide; H2Aub, histone H2A monoubiquitylation; HNSCC, head and neck squamous cell carcinoma; IFN-γ, interferon-gamma; IGF-I, insulin-like growth factor-I; IKE, imidazole ketone erastin; Keap1, Kelch-like ECH-associated protein 1; MCU, mitochondrial calcium uniporter; MDSCs, myeloid-derived suppressor cells; mTORC2, mammalian target of rapamycin complex 2; NAC, N-acetyl cysteine; NADPH, nicotinamide adenine dinucleotide phosphate; Nrf2, nuclear erythroid 2-related factor; OTUB1, ubiquitin hydrolase otubain-1; PDAC, pancreatic ductal adenocarcinoma; PD-L1, programmed death ligand 1; P-gp, P-glycoprotein; PPP, pentose phosphate pathway; PRC1, protein regulator of cytokinesis 1; PUFA, polyunsaturated fatty acid; ROS, reactive oxygen species; SLC7A11 (xCT), solute carrier family 7 member 11; system xc^−^, cystine/glutamate exchange transporter.
